# Smart device-based testing for medical students in Korea: satisfaction, convenience, and advantages

**DOI:** 10.3352/jeehp.2017.14.7

**Published:** 2017-04-24

**Authors:** Eun Young Lim, Mi Kyoung Yim, Sun Huh

**Affiliations:** 1 Division of Educational Evaluation, Korea Institute for Curriculum and Evaluation, Seoul, Korea; 2 Research and Development Division, Korea Health Personnel Licensing Examination Institute, Seoul, Korea; 3 Department of Parasitology and Institute of Medical Education, Hallym University College of Medicine, Chuncheon, Korea; The Catholic University of Korea, Korea

**Keywords:** Computers, Personal satisfaction, Republic of Korea, Medical students, Tablets

## Abstract

The aim of this study was to investigate respondents’ satisfaction with smart device-based testing (SBT), as well as its convenience and advantages, in order to improve its implementation. The survey was conducted among 108 junior medical students at Kyungpook National University School of Medicine, Korea, who took a practice licensing examination using SBT in September 2015. The survey contained 28 items scored using a 5-point Likert scale. The items were divided into the following three categories: satisfaction with SBT administration, convenience of SBT features, and advantages of SBT compared to paper-and-pencil testing or computer-based testing. The reliability of the survey was 0.95. Of the three categories, the convenience of the SBT features received the highest mean (M) score (M= 3.75, standard deviation [SD]= 0.69), while the category of satisfaction with SBT received the lowest (M= 3.13, SD= 1.07). No statistically significant differences across these categories with respect to sex, age, or experience were observed. These results indicate that SBT was practical and effective to take and to administer.

In 2020, the Korean medical licensing examination will be administered as a smart device-based test using a tablet PC as the user interface [1]. Therefore, medical schools have attempted to implement smart device-based testing (SBT) in the administration of practice licensing examinations and have investigated students’ satisfaction with SBT, as well as its effectiveness and convenience, in order to improve students’ adaptation to it. The purpose of this study was to investigate satisfaction with SBT and its perceived convenience among medical students at Kyungpook National University, Korea.

## Study design

This was a cross-sectional observational study.

## Materials and subjects

The evaluation survey included all 108 junior medical students at Kyungpook National University, Korea. They took a practice licensing examination using SBT and filled out an evaluation survey in September 2015. The evaluation survey for SBT consisted of three categories: satisfaction with SBT (two items), convenience of SBT features (13 items), and advantages of SBT (13 items). The category assessing the advantages of SBT was composed of two parts: one compared respondents’ satisfaction with SBT to their satisfaction with paper-and-pencil testing, and the other part contained a similar comparison with computer-based testing (CBT). The following 5-point Likert scale was used for the survey: 1, strongly disagree; 2, disagree; 3, neutral; 4, agree; and 5, strongly agree. Table 1 presents the content of the three categories, the number of items, and the reliability of the survey. For determining the reliability of the survey, internal consistency (Cronbach α) was used. The Cronbach α coefficient of the survey was 0.905, and the range of this coefficient for each item was 0.807 to 0.936; this indicated strong internal consistency and a high level of reliability. Table 2 presents the respondents’ background in terms of gender, age, and experience with CBT and SBT. The percentage of respondents with CBT experience was 96.3%, and the corresponding value for SBT was 17.6%. This indicates that most of the students had some experience with CBT, but not many students had experience with SBT.

## Statistics

The results of the evaluation of SBT were summarized using descriptive statistics in SPSS for Windows ver. 23.0 (IBM Corp., Armonk, NY, USA). The responses regarding satisfaction, convenience, and advantages were compared according to sex, age, experience with CBT, and experience with SBT. Two-way repeated-measures analysis of variance test was conducted for a comparative analysis using DBSTAT ver. 5.0 (DBSTAT Co., Chuncheon, Korea).

## Ethical approval

Informed consent was obtained from the subjects. The study design was approved by the institutional review board of Hallym University (HIRB-2015-092).

Table 3 shows the descriptive statistics of the three categories and the two subcategories regarding the advantages of SBT. The category of the convenience of SBT received the highest mean (M) score (M= 3.75, standard deviation [SD]= 0.69). The next highest mean was that of the category assessing the advantages of SBT compared to paper-and-pencil testing (3.43). The lowest mean was that of the category evaluating satisfaction with SBT (M= 3.13, SD= 1.07). The means among the three categories were significantly different (P< 0.0001). Supplementary files of the coding books and integrated data for each category are presented in Supplement 1. Appendix 1 presents the results of all the items. The range of the 28 items was 2.83 to 4.23. Satisfaction with SBT was assessed using two items: satisfaction with using a tablet PC as a testing tool (M=2.92, SD=1.24) and lack of difficulty in carrying out the testing according to the advice for test-takers presented on the tablet PC (M=3.13, SD=1.26). The percentage of negative responses (strongly disagree and disagree) was higher than 30% for the former item; this implies that further improvement in SBT administration is necessary.

The category evaluating the convenience of SBT features was composed of 13 items. The range of the items in this category was 2.83 to 4.23. The highest mean score (M= 4.23, SD= 0.74) was found for the item assessing whether the function of identifying items that were not marked before submitting the answers was convenient, and the next highest mean score was found for the following item: ‘it was convenient to have a ‘check’ function for later review’ (M=4.18, SD= 0.77). This indicates that students recognized that the features that enabled them to review and correct their answers were the most convenient. The lowest mean was for the item assessing whether it was convenient to use the functions of zoom in and zoom out when viewing an image or replaying a video file (M= 2.83, SD= 1.29). The next lowest mean was of the following item: ‘the loading time was adequate before the start of video playback’ (M= 3.25, SD= 1.29). Therefore, our results indicate that the features related to video need to be improved.

The results for the advantages of SBT compared to paper-and-pencil testing were as follows: The range of mean scores was 2.91 to 3.89, indicating that the advantages of SBT were positively evaluated by the students. The highest mean was for the item assessing whether the absence of separate answer-card marking was helpful in managing the testing time (M= 3.89, SD= 1.06). The next highest mean was for the following item: ‘it was convenient to select the correct answer on the tablet PC screen rather than marking an optical mark reader (OMR) answer card’ (M= 3.85, SD= 1.06). The lowest mean was for the following item: ‘tablet PC-based testing was more convenient than paper-and-pencil testing’ (M= 2.91, SD= 1.09). The next lowest mean was for the following item: ‘it was good to be able to see materials such as photographs, sounds, and video clips’ (M=3.07, SD= 1.09). The range of the advantages of SBT compared to CBT was 2.94 to 3.34. The highest mean was for the following item: ‘there was no noise or heat from the desktop computer, so the examination environment was comfortable’ (M= 3.34, SD= 1.02). The lowest mean was for the following item: ‘the testing instrument was smart, and it helped me to focus more on the testing’ (M=2.94, SD=1.14).

**Fig. 1.**
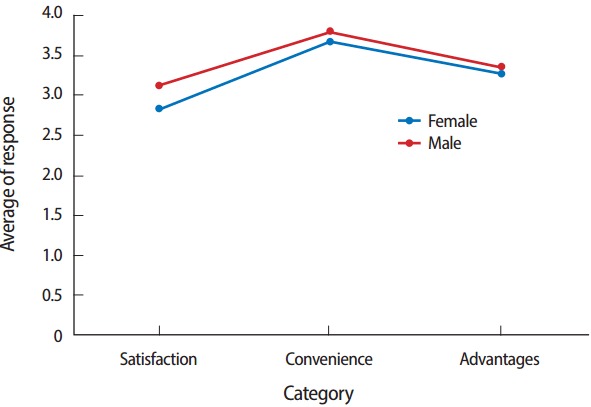
Average responses in each category on a 5-point Likert scale on items assessing satisfaction with smart device-based testing, as well as its convenience and advantages, among medical students in Korea according to gender.

No statistically significant differences were observed in the mean scores for satisfaction with SBT, its convenience, and its advantages according to sex (P=0.2979), age (P=0.7126), experience with CBT (P= 0.5024), or experience with SBT (P= 0.2640) (Fig. 1).

SBT has also been administered in several other medical schools in Korea, where it has been known as ubiquitous-based testing (UBT). UBT was established to be a satisfactory and convenient testing tool in one of these medical schools [2]. In another medical school, students had a positive attitude towards the UBT interface. The factors of having experience with CBT and being male were associated with favorable responses towards UBT [3]. In this study, students thought that SBT could improve the effectiveness of taking a test, reduce extra work such as marking answers on an OMR sheet, and make the testing environment more pleasant than that in the computer lab. As the means of the items showed positive patterns for the satisfaction and convenience of SBT, most items had a high rate for ‘agree’ and ‘strongly agree.’ In conclusion, the results of the survey indicate a positive attitude toward SBT. However, the technical features of SBT, such as the zoom in and zoom out functions when viewing an image and playing video clips, could be improved further.

## Figures and Tables

**Table 1.** The contents, number of items, and Cronbach α coefficient of each category in the survey

Categories	Content	No. of items	Cronbach a
Satisfaction of SBT	Satisfaction of students taking SBT	2	0.807
Convenience of SBT features	Degree of convenience of SBT features	13	0.916
Advantages of SBT	Advantage of SBT compared to a paper testing and pencil testing and a computer based testing.	13	0.936
	1) Compared to a paper and pencil testing	7	0.905
	2) Compared to a computer based testing	6	0.892
Total		28	0.905

SBT, smart device-based testing.

**Table 2.** The number of subjects based on their background

Background	Frequency (%)
Gender	
Male	70 (64.8)
Female	38 (35.2)
Total	108 (100.0)
Age (yr)	
20-24	2 (1.9)
25-30	93 (86.1)
≥31	13 (12.0)
Total	108 (100.0)
Experience of taking computer-based testing	
No	4 (3.7)
Yes	104 (96.3)
Total	108 (100.0)
Experience of taking smart device-based testing	
No	89 (82.4)
Yes	19 (17.6)
Total	108 (100.0)

**Table 3.** The descriptive statistics of 3 categories and 2 subcategories (N = 108)

	Mean ± standard deviation
Satisfaction of SBT	3.13±1.07
Convenience of SBT	3.75±0.69
Advantages of SBT	3.33±0.83
1) Compared to a paper and pencil testing	3.43±0.89
2) Compared to a computer based testing	3.20±0.87

SBT, smart device-based testing.

## Supplementary materials

Supplement 1. Data file is available from https://dataverse.harvard.edu/dataset.xhtml?persistentId=doi:10.7910/DVN/GT2IZY

Supplement 2. Audio recording of the abstract.

## Appendix 1. Frequency and mean of each item in the survey given to medical students in Korea after smart device-based testing in 2015

**Table:**

Item	Mean ± standard deviation	Frequency				
		Strongly disagree	Disagree	Neutral	Agree	Strongly agree
Satisfaction with SBT						
1. I was satisfied with using the tablet PC as the testing tool.	2.92 ±1.24	19	20	30	29	10
2. There was no difficulty in carrying out the test according to the advice for test takers presented on the tablet PC.	3.13 ±1.26	15	18	20	30	16
Convenience of SBT						
3. Function of the remaining testing time notification was more convenient than an announcement of the remaining testing time.	3.35 ±1.26	11	19	20	37	21
4. The selection and correction function of the correct answer was convenient.	3.80 ±1.00	3	10	19	50	26
5. It was convenient to see one item at a time on the screen.	3.85 ±1.01	2	11	19	45	31
6. The function of identifying items that were not marked before submitting the answers was convenient.	4.23 ±0.74	0	3	11	52	42
7. It was convenient to be able to see the previous items, the next items, and all items.	3.97 ±0.86	2	4	17	57	28
8. It was convenient to be able to see the solved items, unsolved items, checked items, and the items noted for later review selectively	3.91 ±1.03	3	9	17	45	34
9. It was convenient to have a 'check'function for later review.	4.18±0.77	0	3	15	50	40
10. The'check wrong answers'function to check the wrong answer option was convenient.	3.95 ±0.95	1	7	24	40	36
11.The font size was appropriate.	3.85 ±0.96	4	6	16	58	24
12.The typeface was appropriate.	3.93 ±0.85	3	2	19	60	24
13. The loading time was adequate before the start of video playback.	3.25 ±0.94	5	12	55	26	11
14. It was convenient to use then functions of zoom in and zoom out when viewing an image and replaying a video file.	2.83 ±1.29	22	22	27	26	11
15.The overall screen configuration was appropriate.	3.58 ±0.91	4	6	34	51	13
Advantages of SBT						
Comparison with paper-and-pencil testing						
16. It was convenient to select the correct answer on the tablet PC screen rather than marking the optical mark reader answer	3.85 ±1.03	5	6	18	50	29
17. The absence of separate answer-card marking was helpful in managing the testing time.	3.89 ±1.06	5	6	19	44	34
18.The psychological burden (tension) decreased because there was no separate answer-card marking.	3.75±1.16	7	10	17	43	31
19.The font size was large and clear, minimizing eye fatigue.	3.35 ±1.08	9	10	36	40	13
20. It was good to be able to see materials such as photographs, sounds, and video clips.	3.07 ±1.09	11	18	40	30	9
21.Tablet PC-based testing was more convenient than paper-and-pencil testing.	3.21 ±1.25	14	18	21	41	14
22.The test taker's ability is assessed more accurately by tablet PC-based testing than by paper-and-pencil testing.	2.91 ±1.09	16	16	43	28	5
Comparison with desktop-based testing						
23.The touch-screen method was more convenient than the mouse-click method for selecting the correct answer.	3.33 ±1.12	10	12	31	42	13
24. There was less eye strain in the case of the tablet PC than in the case of the desktop PC monitor.	3.22 ±1.09	9	14	42	30	13
25. There may be a decrease in cheating behavior during tablet PC-based testing as compared to desktop PC-based testing.	3.24 ±1.03	9	10	45	34	10
26. The testing instrument was smart, and it helped me to focus more on the test.	2.94 ±1.14	16	15	46	21	10
27. There was no noise or heat from the desktop computer, so the examination environment was comfortable.	3.34 ±1.02	8	11	34	46	9
28. Tablet PC-based testing is an improved testing method compared to the existing desktop PC-based testing method.	3.12 ±1.09	10	18	39	31	10

SBT, smart device-based testing.
